# Eosinophilic esophagitis: a matter of flavoring

**DOI:** 10.1186/2045-7022-5-S3-P161

**Published:** 2015-03-30

**Authors:** Raquel Gomes, Carmo Abreu, Carlos Loureiro, Ana Todo-Bom

**Affiliations:** 1 Centro Hospitalar e Universitário de Coimbra, Coimbra, Portugal; 2 Centro Hospitalar do Porto, Porto, Portugal

## 

Eosinophilic esophagitis (EoE) is an inflammatory disease characterized by eosinophilic infiltration of the esophageal wall. It is an increasingly recognized disease that affects mostly caucasian males in two peaks of incidence: 5-10/20-40 years. Studies show that more than 90% of this patients are sensitized to mite or polens, 50% to food, and that the contact with them exacerbates the disease.

We aim to present a 30 years old caucasian male patient with a previous history of rhinitis, sent in 2012 to our immunoallergology department by urticaria, intermittent dysphagia and food impactation for 10 years. First impactation occurred in 2008 and the upper gastrointestinal endoscopy (UGIE) revealed annular strictures, longitudinal grooves and the respective biopsy showed eosinophilic inflammatory infiltrate. In the Gastroenterology department started a proton pump inhibitor and later topical corticosteroids for 3 months. In January 2012 after the intake of dried pumpkin seeds the patient had an episode of generalized urticaria plus facial angioedema without other systemic involvement; this episode occurred again after consumption of dried flavored cashews. In August 2012 a new food impactation occurred.

(1) Skin prick tests (mm):

- Food and spices: curry 10; mustard 10; paprika 6; cumin 3

- Aeroallergens: Histamine 4; birch 3; grass polen 6; *Dermatophagoides pteronyssinus* (Dp) 8; *Lepidoglyphus destructor* (Ld) 15.

- Prick-to-prick tests (mm): paprika 16; curry 15; cinnamon 15; mustard 14; saffron 7

(2) Laboratory tests:

- Normal blood count, protein concentrations, renal and liver function

- IgG 15.3g/dL, IgA 3.1g/dL, IgM 0.47g/dL, IgE 1440Ul/ml

- Specific IgE (kU/L): *Dactylis glomerata* 47,1; *Phleum pratense* 19,2; *Betula verrucosa* 2,52; Dp 53,4 e Ld 8,7

(3) UGIE (2012): Shatski ring and ulceration downstream. Biopsies: eosinophilic infiltrate (40cel/high-powered field), microabscesses of the wall

He began avoid those condiments/spices and use topical corticosteroids (TC) for esophageal mucosa, basic mite eviction and hygiene measures. He remained asymptomatic and 3 months after stopping the TC, a control UGIE was performed. It showed no macroscopical changes and biopsy revealed a reduction of eosinophilic infiltration (15cel/HPF). With this case report we intend to highlight the importance of recognizing EoE and identify exacerbating factors considering the importance of the avoiding measures in the disease control.

## Consent

Written informed consent was obtained from the patient for publication of this abstract and any accompanying images. A copy of the written consent is available for review by the Editor of this journal.

